# A Genome Wide CRISPR Pro**fi**ling Approach Identi**fi**es Mechanisms of Cisplatin Resistance in Head and Neck Squamous Cell Carcinoma

**DOI:** 10.21203/rs.3.rs-3922565/v1

**Published:** 2024-02-20

**Authors:** Megan Ludwig, Andrew Birkeland, Joshua Smith, Elizabeth Gensterblum-Miller, JIngyi Zhai, Aditi Kulkarni, Hui Jiang, Chad Brenner

**Affiliations:** University of Michigan–Ann Arbor; University of Michigan–Ann Arbor; University of Michigan–Ann Arbor; University of Michigan–Ann Arbor; University of Michigan–Ann Arbor; University of Michigan–Ann Arbor; University of Michigan–Ann Arbor; University of Michigan–Ann Arbor

**Keywords:** Head and neck squamous cell carcinoma, HNSCC, NOTCH, Notch1, CRISPR, cisplatin, resistance, sensitivity, knockout

## Abstract

**Background:**

Head and neck squamous cell carcinoma (HNSCC) is a lethal disease with poor survival rates, especially for cancers arising in the oral cavity or larynx. Cisplatin is a key chemotherapeutic for HNSCC; however poor survival rates may be partially due to cisplatin resistance observed in some HNSCCs. Here, we examined the utility of genome-wide CRISPR knockout profiling for nominating pivotal mechanisms of cisplatin resistance in HNSCC models.

**Methods:**

We characterized the cisplatin sensitivity of 18 HNSCC cell lines. Next, we used a genome-wide CRISPR/Cas9 library to identify genes involved in cisplatin resistance. We next performed validation assays in the UM-SCC-49 cell line model.

**Results:**

Our data prioritized 207 genes as pivotal for cisplatin resistance in HNSCC, including novel genes *VGLL3, CIRHA1, NCOR1, SPANXA1, MAP2K7, ULK1,* and *CDK16*. Gene set enrichment analysis identified several *NOTCH* family genes comprising the top pathway driving cisplatin resistance, which we then validated using a targeted *NOTCH1* knockout model. Interestingly, we noted that HNSCC models with natural NOTCH pathway alterations including single allele mutations and/or frameshift alterations had diverse responses to cisplatin treatment suggesting that complex and multi-faceted mechanisms contribute to cisplatin resistance in HNSCC.

**Conclusions:**

Collectively, our study validates a genome-wide CRISPR/Cas9 approach for the discovery of resistance mechanisms in HNSCC, adds to the growing evidence that *NOTCH1* status should be evaluated as a biomarker of cisplatin response and provides a framework for future work aimed at overcoming cisplatin resistance.

## Introduction

Head and neck squamous cell carcinoma (HNSCC) is the sixth most common cancer worldwide, with more than 50,000 new cases diagnosed annually in the United States alone [1]. Morbidity and mortality of HNSCC remains high, and as many as 50% of patients who present with advanced stage HNSCC of the oral cavity or larynx will develop recurrent disease within the first two years after treatment [2]. Platinum-based chemotherapy (primarily cisplatin) remains the backbone of systemic therapy for HNSCC in the definitive, adjuvant, and recurrent and metastatic settings [3–5]. However, innate or acquired resistance to platinum-based chemotherapy remains a pivotal clinical problem, particularly in the recurrent and metastatic setting [6, 7]. Currently, no biomarkers exist that can predict rate and durability of response to platinum-based chemotherapy in HNSCC. We posit that such predictive biomarkers would dramatically improve our ability to tailor precision therapy regimens for patients with HNSCC in all disease settings.

Because of the significance and complexity of this clinical problem, several research teams have used a variety of approaches to identify mechanisms of resistance to platinum-based chemotherapy in HNSCC cell line models and patient-derived xenografts. These have included specific mechanistic studies in cell line models [8], genome-wide shRNA knockdown approaches [9], and next generation sequencing (NGS) of primary tumors [10]. These studies have identified important and diverse mechanisms of platinum resistance such as NF**k**B signaling [11], TRK signaling [12], and excision repair mechanisms [13]. However, no study has yet identified highly recurrent resistance pathways or common effectors of such pathways that may have broad clinical impact for patients. In recent years, genome-wide CRISPR libraries have become powerful tools for identifying essential genes [14] and key drivers of therapeutic sensitivity [15, 16]. These genome-wide knockout technologies have not yet been applied to nominate mechanisms of platinum resistance in HNSCC. We hypothesize that genome-wide approaches may provide an opportunity to broadly characterize the many diverse drivers of platinum resistance and enable the identification of convergent pathways that may be useful as predictive biomarkers and/or therapeutic targets.

Here, we sought to optimize and validate a strategy to identify mechanisms of cisplatin resistance using CRISPR libraries in HNSCC cell line models. We hypothesized that the integration of CRISPR profiling approach with gene set enrichment analysis would permit prioritization of functionally pivotal pathways that drive cell survival following cisplatin treatment. Thus, we leveraged our comprehensively characterized UM-SCC cell line panel representing a diverse population of HNSCC to prioritize mechanisms of cisplatin resistance that may have clinical value for patients with HNSCC.

## Methods

### Cell Culture

Cell lines were cultured in Dulbecco’s Modified Eagle’s Medium (DMEM) (Invitrogen #11965) containing 10% fetal bovine serum (FBS, Sigma), 1% NEAA (Invitrogen 15140122) and 7 μL/mL penicillin-streptomycin (Invitrogen 15140122) in a humidified atmosphere of 5% CO_2_ at 37°C, as described [17]. Cells were tested for mycoplasma contamination using the MycoAlert detection kit (Lonza) and genotyped, as described [18]. UM-SCC lines were transduced with the Human Genome-wide CRISPR KnockOut (GeCKO) pooled library, version 2A which was a gift from Feng Zhang [15]. Conditions for transduction were established for a multiplicity of infection (MOI) of 30%. After 7 days of puromycin selection, the cells were expanded and seeded per treatment. To preserve at least 300x coverage, 30 million cells were seeded per treatment for the GeCKO libraries. At the end of treatment, DNA was extracted from the remaining cells using Gentra Puregene Cell Kit (Qiagen), as described [19]. For cisplatin treatment, cells were dosed with 0.125 μM cisplatin (Selleckchem S1166) or DMSO (Sigma Aldrich) for 24 hours, once a week for two weeks.

### GeCKO Library Preparation

To preserve coverage of the GeCKO library, 130 μg of DNA was used to PCR amplify the gRNA sequence using the Herculase II Fusion DNA Polymerase (Agilent # 600675). 13 reactions with 10 μg input DNA was amplified with the following primers:

PCR #1 Fwd: AATGGACTATCATATGCTTACCGTAACTTGAAAGTATTTCG

PCR #2 Rev: GGTCTTGAAAGGAGTGGGAATTGGCTCCGGTGCCCGTCAG

The 13 reactions were combined, and then 5 μL were used to set up the second round PCR reactions with the following primers:

PCR #2 Fwd: AATGATACGGCGACCACCGAGATCTACACTCTTTCCCTACACGACGCTCTTCCGATCT(1–9bp stagger)AAGTAGAGtcttgtggaaaggacgaaacaccg

PCR #2 Rev: CAAGCAGAAGACGGCATACGAGATTCGCCTTAGTGACTGGAGTTCAGACGTGTGCTCTTCCGATCTataacggactagccttattttaac

The uppercase sequence represents Illumina adapters. The forward primer has the TruSeq Universal Adapter, and the reverse primer consists of Illumina P7, 8bp index, and multiplexing PCR primer 2.0. The underlined sequence represents an 8bp barcode. Lowercase letters are the priming sites for the lentiviral construct.

The PCR product was gel extracted and purified using Gel Extraction PCR Purification Kit (Qiagen) as described [20] before submission to the University of Michigan Advanced Genomics Core for sequencing with the Illumina MiSeq V3 Kit.

### Analysis of CRISPR Libraries

Reads were demultiplexed by barcode and then mapped to the corresponding reference library using an in-house Python script. gRNA counts were input into Model-based Analysis of Genome-wide CRISPR/Cas9 knockouts (MAGeCK, v0.5.2) [21]. MAGeCK algorithms calculated significant gRNAs and genes, and genes with an α-RRA score of ≤ 0.005 and at least two ‘good gRNAs (‘good’ as established by the MAGeCK software) were advanced to gene set enrichment analysis (GSEA). Gene sets were then uploaded into GSEA (MIT, Broad) to identify significant overlap with Hallmark, KEGG and GO biological process pathways with FDR q-value< 0.05 considered significant, as described [22].

### Generation of Clonal Knockout Model

UM-SCC-49 was transduced with a lentiviral CRISPR construct targeting *NOTCH1* (Sigma Aldrich, HS0000408729) and after antibiotic and GFP selection, individual clones were isolated. DNA was extracted from clones (Qiagen, Gentra Puregene Cell Kit) and the gRNA region amplified by PCR using Platinum HiFi Taq (Invitrogen). Primers for amplification are shown in **Supplemental Table 1**. PCR products were then ligated into pCR8 vector (ThermoFisher, K250020), transformed, and plasmid DNA extracted from individual colonies (Qiagen, QIAprep Spin Miniprep Kit), as described [23], and submitted for Sanger sequencing at the University of Michigan Advanced Genomics Core. Sequences were aligned using the DNASTAR Lasergene software suite.

### Immunoblotting

Western blot analysis was performed as previously described [24, 25]. Briefly, UM-SCC cell lines at 70–80% confluency were rinsed with PBS and lysed in buffer (150 mM NaCl, 10% Glycerol, 1% NP40, 0.1% Triton X-100, 1 mM PIPES, 1 mM MgCl, 50 mM Tris) containing protease and phosphatase inhibitors (Thermo 186129, 1861277). See **Supplemental Table 2** for primary and secondary antibodies used.

### Transcriptome Sequencing and Analysis

Transcriptome analysis was performed on 1 ug of total RNA isolated using the Qiagen RNeasy Mini Kit (Cat No: 74106) using Illumina stranded transcriptome library preparation kits and subsequent 300 nucleotide paired end sequencing to > 100x depth on an Illumina HiSEQ4000 for UM-SCC-49 and UM-SCC-49 *NOTCH1* knockout cells [26]. Library generation and sequencing were performed at the University of Michigan Advanced Genomics Core, and no quality issues were identified in the generation of sequencing data. To calculate gene expression in fragments per kilobase million (FPKM), we aligned reads using STAR (v2.5.3a) according to the standard two-step alignment process and then processed the data with Cufflinks (v2.2.1). Using the FPKM read counts, we then defined gene signatures that were >2-log2 fold upregulated or downregulated in the knockout model relative to the control and uploaded the gene sets into GSEA (MIT, Broad) to identify significant overlap with Hallmark, KEGG and GO biological process pathways with FDR q-value< 0.05 considered significant.

### Clonogenic Cell Survival Assays

Cultured cells were dosed with cisplatin for 24 hours at the indicated dose and were then plated in triplicate and allowed to grow for two weeks, before being fixed and stained with 6% glutaraldehyde/0.5% crystal violet. Colonies with greater than 50 cells were counted and percent survival was calculated as number of colonies divided by number of plated cells. Survival fraction was calculated by dividing treatment cells by untreated controls.

## Results

**Figure 1:**
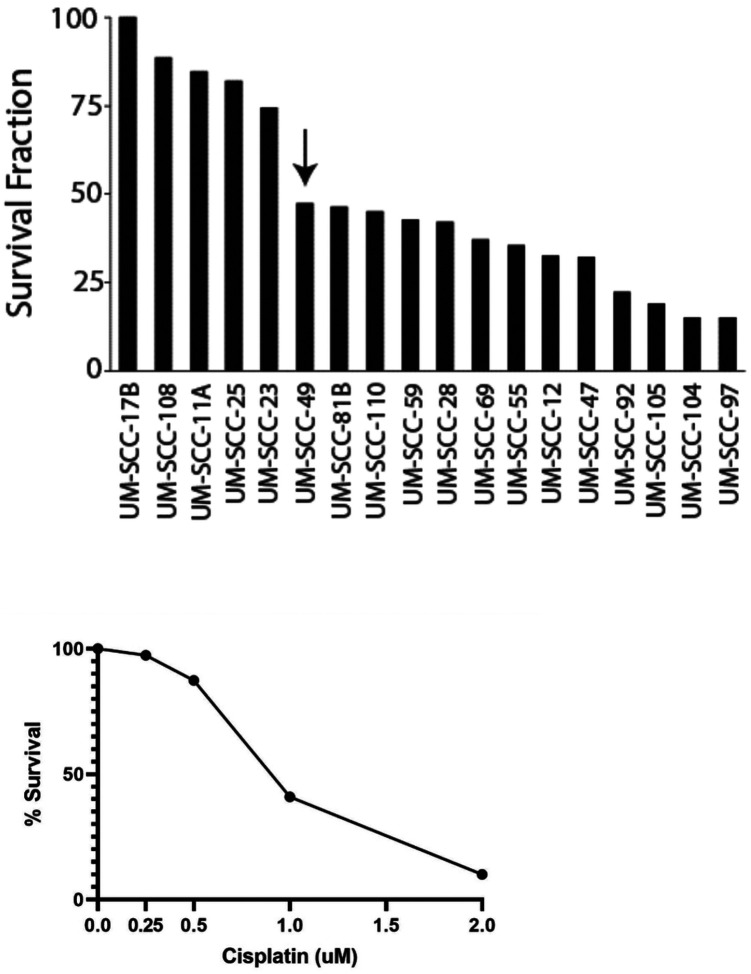
Characterization of Cisplatin resistance in HNSCC cell line models. **A)** Clonogenic cell survival fraction of 18 UM-SCC cell lines following treatment with 1 μM cisplatin. UM-SCC-49, indicated by arrow, was selected for functional genomics screening based on an intermediate response to cisplatin. Experiments completed in duplicate for each model. **B)** Percent survival of the UM-SCC-49 cell line after treatment with multiple doses of cisplatin as determined in **A**. Each dot represents a dose tested, with a line drawn for interpretation.

We first sought to characterize the relative sensitivity of several of our UM-SCC cell line models to cisplatin. We treated our cell lines with 1 μM cisplatin for 24 hours then quantified the survival fraction 10–14 days later (as optimized for the clonogenic efficiency of each cell line). In total, we characterized the cisplatin sensitivity of 18 UM-SCC cell lines including 10 derived from oral cavity primaries and 8 derived from larynx primaries [25, 27]. The panel contained 15/18 (83%) HPV-negative cell lines. The remaining three were positive for HPV Type-16 (UM-SCC-47, UM-SCC-104) and HPV Type-18 (UM-SCC-105) (Fig. 1). Ultimately, UM-SCC-17B, UM-SCC-108 and UM-SCC-11A were highly resistant to this cisplatin dose, while UM-SCC-49, UM-SCC-81B and UM-SCC-110 showed intermediate sensitivity. As expected, the HPV + cell lines were the most sensitive to cisplatin treatment.

**Figure 2:**
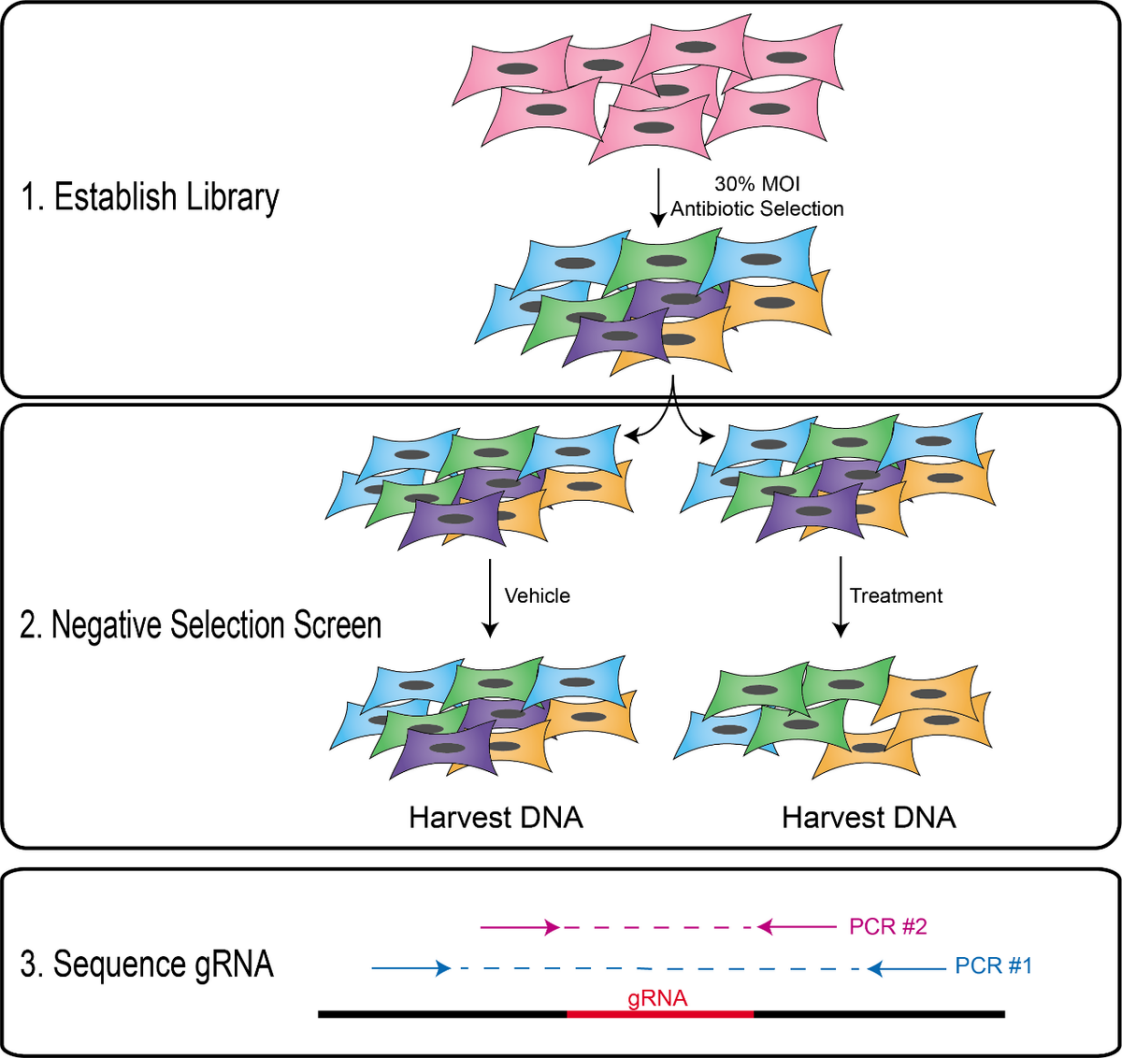
Schematic of negative selection screen from CRISPR library. First, a pooled knockout library is established, taking a wild type cell line and adding the lentiviral library at 30% MOI and then undergoing antibiotic selection. Here, different knockouts from gRNAs are represented with different colors. Then, the CRISPR library pool is expanded and split for treatment with either vehicle control or cisplatin. At the end of treatment, DNA is harvested from the surviving populations, and NGS libraries are prepared using a nested PCR setup flanking the gRNA. The gRNAs in each population are then quantified by NGS.

With our primary goal being to validate an approach to nominate drivers of cisplatin resistance in HNSCC, we chose to advance a negative selection screening model using the publicly available GeCKO CRISPR library [15, 28]. Given the wide range of cisplatin responses in our models, we chose to advance UM-SCC-49 for our functional genomics experiment based on both the intermediate response to cisplatin as well as the positive growth characteristics of the cell line in roller bottles required for the profiling of >300 million cells (data not shown). The schematic in Fig. 2 outlines the creation, expansion, and experimental design to identify genetic knockouts that result in sensitivity to cisplatin, and as such nominate potential novel therapeutic combinations for effective cancer cell death. Thus, we transduced UM-SCC-49 cells with the GeCKO v2A CRISPR library, then selected and expanded this pool before treating with cisplatin or vehicle control. At the end of treatment, we isolated genomic DNA from the remaining cell pools and used Illumina-based NGS to quantify the gRNAs in each population.

**Figure 3:**
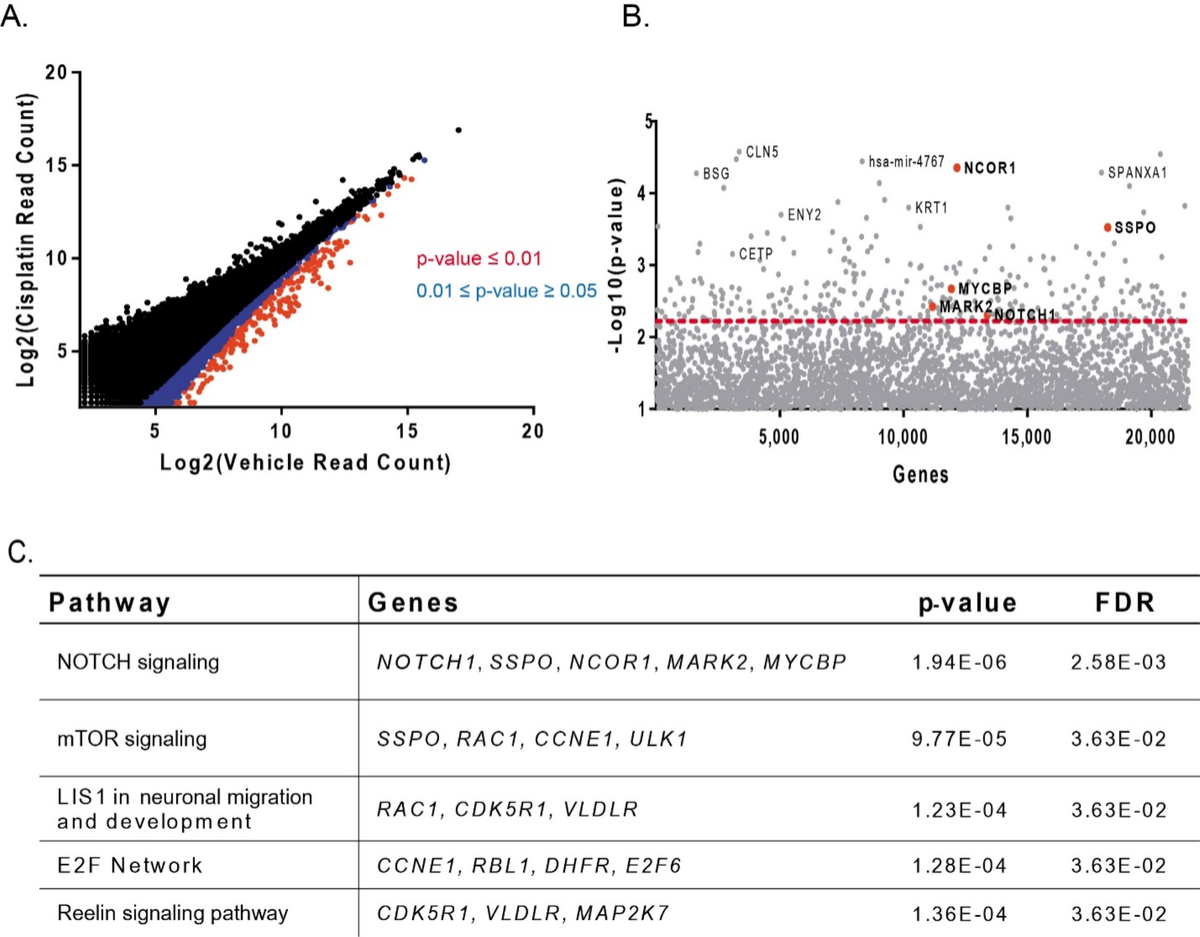
CRISPR profiling and GSEA prioritizes cisplatin resistance pathways in UM-SCC-49. A) gRNA read counts plotted for cisplatin vs vehicle control treatment. gRNAs in blue (0.01 ≤ p-value ≤ 0.05) and red (p-value ≤ 0.01) indicate significant depletion. B) The −log10(p-value) for each gene is plotted, where the p-value represents the significance of depletion in the cisplatin treatment compared to the vehicle control. A dotted line representing a p-value cutoff of 0.005 is shown, where all genes above the line have a p-value ≤ 0.005. Significantly depleted genes in the Notch pathway are bolded. C) GSEA results for genes with a p-value ≤ 0.005, where the enriched pathway and significantly depleted genes are noted as well as p-value and FDR from the GSEA analysis.

To perform quality control analysis on our NGS results, we first evaluated the representation of the complete library in our final cell pools. Importantly, we observed that over 80% of the original GeCKO v2A library was represented in both control and treatment populations (**Supplemental Fig. 1**), suggesting that the library diversity was maintained throughout cisplatin challenge. We then compared the gRNA sequences from the cisplatin treatment to the vehicle control using the MAGeCK algorithm for CRISPR knockout screens.^21^ Our analysis pipeline identified specific gRNAs (Fig. 3A, **Supplemental Table 3**) and genes (Fig. 3B, **Supplemental Table 4**) that were significantly depleted with cisplatin treatment compared to vehicle control. In total, 207 significant genes were identified in the analysis. Top genes with highest significance were *VGLL3, CIRH1A, NCOR1,* and *SPANXA1* - suggesting these genes may have a pivotal role in promoting cisplatin resistance in HNSCC. Of these genes, *VGLL3* encodes a transcription factor whose signaling network is not well-defined, but whose expression has been suggested as a prognostic biomarker in gastric cancer [29]. *CIRH1A* encodes a ribosomal protein implicated in proliferation of colorectal cancer cells [30]. *NCOR1* encodes a nuclear corepressor that has wide ranging functions in many cellular processes including metabolism, inflammation, genomic stability and carcinogenesis [30]. *SPANXA1* is part of the SPANX family of cancer-testis antigens expressed in normal testes but dysregulated in various cancers including HNSCC and lung adenocarcinoma [31, 32]. Other significant genes of interest that were identified in our screen included *MAP2K7, ULK1* and *CDK16. MAP2K7* is a member of the MAPK family that activates JNK signaling. *ULK1* encodes a kinase in the mTOR signaling cascade involved in regulating autophagy [33]. Finally, *CDK16* is a more recently identified member of the CDK gene family and has also been identified as promoting resistance to radiation in lung cancer [34].

After completing our gene level analysis, we then advanced significant genes (p-value ≤ 0.005) for GSEA analysis which nominated several pathways as candidate drivers of cisplatin resistance in HNSCC (Fig. 3C). Notably, the most significant pathway enriched in the gene set was the *NOTCH* signaling pathway. Genetic knockouts in the *NOTCH* signaling pathway including *NOTCH1, SSPO, NCOR1, MARK2,* and *MYCBP* were significantly underrepresented in the UM-SCC-49 GeCKO pool following cisplatin treatment. Importantly, our previous exome analysis of the UM-SCC-49 cell line demonstrated that these *NOTCH* pathway genes were all wild type in this cell line, making this a strong model for assessing gene knockout effects on cisplatin sensitivity [25]. Furthermore, previous studies in ovarian and colorectal cancer have found that inhibition of the *NOTCH* signaling pathway sensitizes cell line models to cisplatin, which suggested that our genome wide screening method could accurately identify drivers of cisplatin resistance in HNSCC [35, 36].

**Figure 4:**
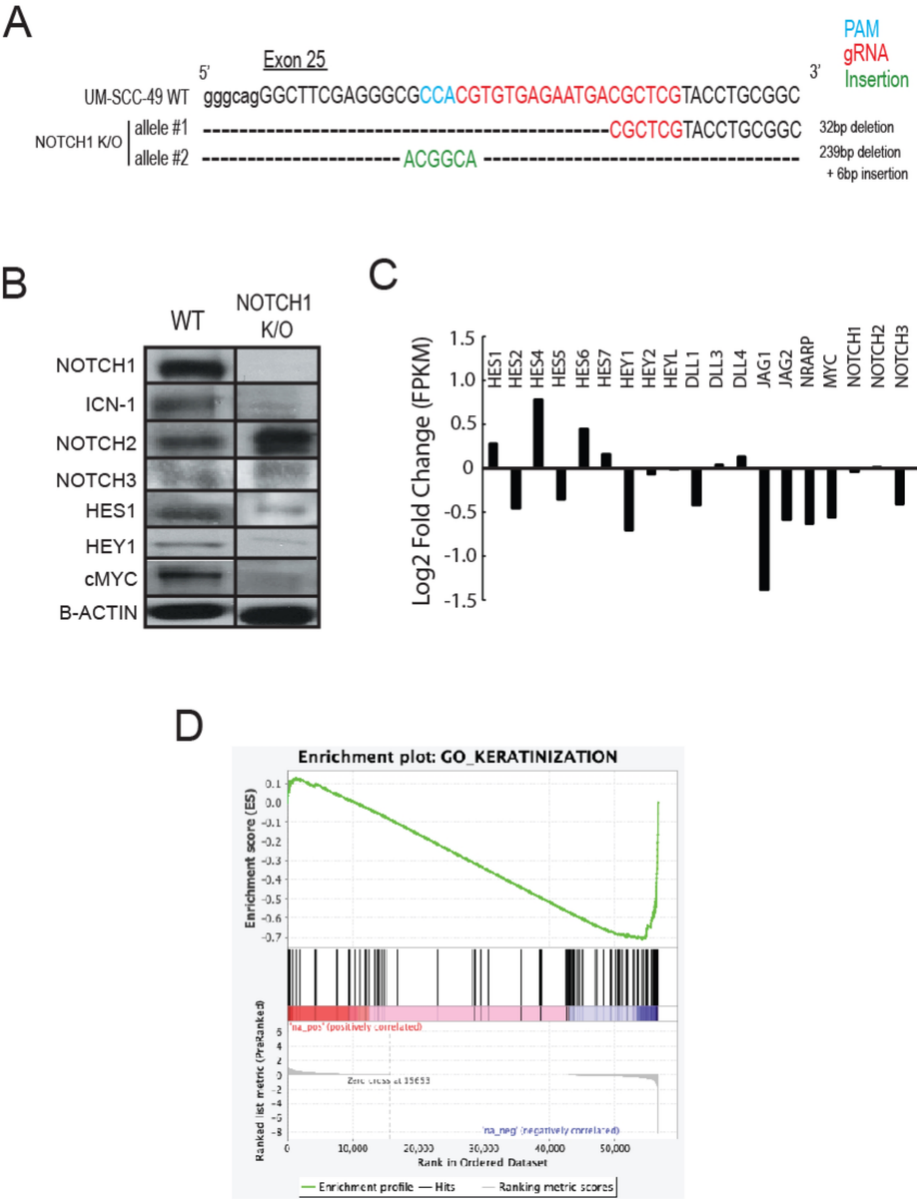
Establishment of *NOTCH1* knockout UM-SCC-49 cell line. A) Schematic of Sanger sequencing results from *NOTCH1* K/O, showing a 32bp deletion and 239bp deletion + 6bp insertion for both allelic copies of *NOTCH1*. The gRNA (red) and PAM sequence (blue) were in exon 25 of *NOTCH1* B) Western blot images of UM-SCC-49 (WT) and *NOTCH1* knockout model. Note that bands shown are from different parts of the same gel or different gels based on specific protein assayed. C) Fold change in fragments per kilobase million (FPKM) expression of several canonical NOTCH pathway effectors from comprehensive transcriptome sequencing of the wild-type and *NOTCH1* knockout model. D) GSEA was performed using the differential gene expression rank list comparing the RNAseq data from the UM-SCC-49 wild-type and *NOTCH1* knockout.

To further validate that knockout of Notch signaling sensitizes UM-SCC-49 cells to cisplatin treatment, we used a targeted CRISPR/Cas9 approach to knockout *NOTCH1* in UM-SCC-49, where the gRNA was targeted to exon 25 as shown in Fig. 4A. Our previous SNP array copy number analysis data demonstrated that the UM-SCC-49 cell line had two copies of wild-type *NOTCH1* [25]. Consistent with this data, we generated two unique frameshift alleles in our *NOTCH1* knockout model, resulting in no detectable expression of Notch1 protein by immunoblot (Fig. 4B). To test the functional impact of *NOTCH1* knockout on baseline activity of the Notch1 signaling pathway in this model, we then assayed protein expression changes of canonical downstream effectors including HES1, HEY1, and cMYC. This demonstrated a significant decrease in effector expression. We also noted that the *NOTCH1* knockout clone had a reciprocal increase in expression of Notch2 protein, and given the decrease of canonical pathway effector expression, we hypothesized that Notch2 may partially compensate for critical functions of Notch1 in this clone.

Because we observed significant differences in protein expression of Notch1 pathway effectors between our models in the absence of additional Notch1 pathway ligand (e.g., DLL1 or JAG1), we then performed RNAseq analysis to define differential transcriptome signatures without ligand stimulation. This analysis demonstrated a decrease in the RNA expression of several canonical Notch1 pathway genes including *HES2, HES5, HEY1, JAG1* and *MYC,* but also showed that *NOTCH1* RNA expression was unchanged (Fig. 4C, **Supplemental Table 5**). This suggested that our knockouts induced frameshift alterations that did not alter *NOTCH1* gene transcript expression, but consistent with the Western blot data, were sufficient to deregulate functional Notch1 protein in the model. Interestingly, we did not observe any changes to *NOTCH2* RNA expression, suggesting that the mechanism driving reciprocal increased Notch2 protein occurs at the level of either RNA translation or protein degradation. Further GSEA demonstrated a significant enrichment of genes from the Go_keratinization gene set (FDR q-value< 0.05, Fig. 4D). This data is consistent with previous reports showing an essential role for Notch1 signaling in keratinization, suggesting that our new knockout cell line functionally replicates mouse models with loss of *NOTCH1* in the epithelium [37, 38].

**Figure 5:**
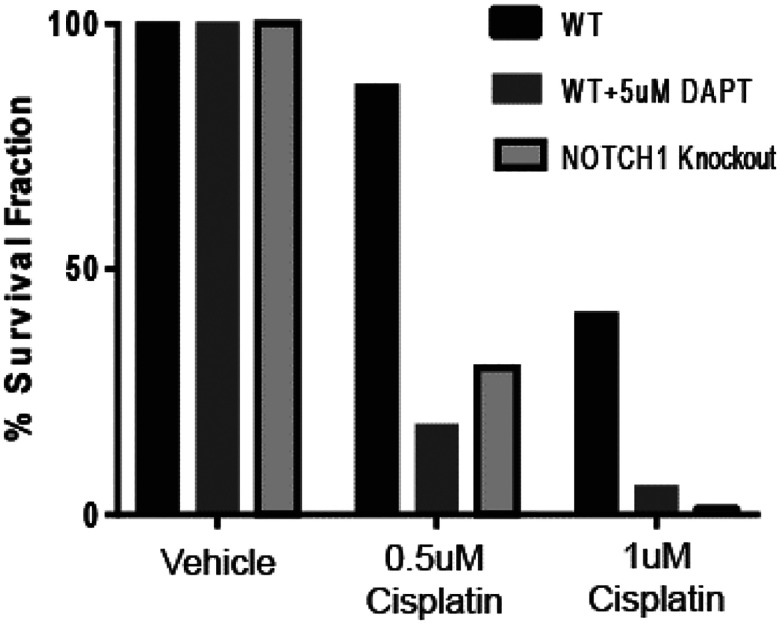
*NOTCH1* inhibition drives cisplatin sensitivity in UM-SCC-49. Bar graph shows percent clonogenic cell survival fraction of UM-SCC-49 WT (black), UM-SCC-49 WT plus 5 μM DAPT treatment (grey), and *NOTCH1* knockout (light grey with black outline) with vehicle control or cisplatin treatment. Experiments repeated in triplicate.

Having confirmed the development of a new *NOTCH1* knockout HNSCC model, we then sought to test if the *NOTCH1* knockout cell line was more sensitive to cisplatin than wild type UM-SCC-49 cells as predicted by our genome wide CRISPR library screen. Subsequent clonogenic cell survival assays showed that *NOTCH1* knockout UM-SCC-49 was significantly more sensitive to cisplatin than wild type UM-SCC-49 cells (Fig. 5). In parallel, we also tested inhibition of Notch1 signaling with the γ-secretase inhibitor DAPT and demonstrated a similar phenotype of cisplatin sensitivity in treated wild-type UM-SCC-49 cells (Fig. 5). Collectively, these parallel experiments demonstrated that Notch1 signaling is a critical mediator of cisplatin resistance in UM-SCC-49 cells.

## Discussion

Here, we present the results of a negative selection CRISPR screen with a cisplatin resistant UM-SCC cell line as well as the subsequent validation of a prioritized pathway from the screen. Our results nominate candidate genes that potentially drive resistance to cisplatin treatment in HNSCC and validates a functional genomics approach that can be used to study cisplatin resistance in genetically diverse models. Our genome-wide CRISPR screen identified the *NOTCH* signaling pathway as the most significant pathway that, when inhibited, sensitized our cell line model to cisplatin treatment. Importantly, these data are consistent with previous results in other cancer models [35, 36] and are particularly interesting for HNSCC given the prevalence of *NOTCH* pathway mutations, which approximate 20% in some series [39, 40]. Our data is also consistent with previous studies that have shown strong correlations between Notch1 protein expression and cisplatin resistance in HNSCC [41–43].

Our study nominates Notch inhibitors as potential combination therapies incorporating cisplatin treatment in biomarker-selected patients [44]. Unfortunately, this combination is unlikely to be feasible in translation, at least with non-specific Notch pathway inhibitors such as γ-secretase inhibitors (GSIs). GSIs have undergone investigations in clinical trials, most notably for patients with activating *NOTCH* mutations in T-cell acute lymphoblastic leukemia [45, 46]. Unfortunately, in these settings GSIs have been observed to have dose limiting gastrointestinal toxicity and have caused growth of both basal cell and squamous cell carcinomas in one clinical trial [47, 48]. However, more recent trials investigating recently developed Notch inhibitors, alone or in combination with a second targeted agent, in advanced solid tumors has shown promising efficacy and safety profiles [46, 49]. Our data demonstrates that Notch1 inhibition is sufficient to sensitize HNSCC cells to cisplatin *in vitro,* suggesting that some of the recently developed Notch1 targeted agents may also sensitize primary and/or recurrent HNSCC tumors to cisplatin in real-world settings [49]. Furthermore, assessment of *NOTCH1* alteration status in HNSCC tumors may permit precision treatment matching for patients with HNSCC, with the potential utilization of cisplatin in *NOTCH1* mutant tumors in induction or definitive chemotherapy regimens with greater success; for example, to account for the variable responses observed in induction chemotherapy in larynx cancers [50].

Our clonogenic data also raise questions of the functional role of specific *NOTCH* gene alterations that are commonly found in primary and recurrent HNSCC. Importantly, our validation data shows that complete knockout of *NOTCH1* or inhibition of Notch1 signaling is sufficient to sensitize cell lines to cisplatin, but that the cell line models characterized in Fig. 1 with variable mutations in *NOTCH* receptors can be highly resistant to cisplatin treatment. For example, UM-SCC-11A harbors an Arg912Trp alteration and UM-SCC-28 a Ser496fs alteration in *NOTCH1,* but are both cisplatin resistant models [25, 51]. As such, it is possible that single allele alterations of *NOTCH1* (or these specific alterations), do not sufficiently disrupt pathway signaling to induce a cisplatin sensitivity phenotype. Alternatively, for example, UM-SCC-25, which is highly cisplatin resistant, harbors both Glu488Ala and Val489fs alterations in *NOTCH1,* Ser879Phe alteration in *NOTCH2* and Tyr258Ter alteration in *NOTCH3* [25, 51]. Thus, despite the high level of *NOTCH* pathway disruption in this model, we may expect that alternative mechanisms contribute to cisplatin resistance in UM-SCC-25 specifically.

Collectively, our data are consistent with recent reports in the literature highlighting a pivotal role for *NOTCH* pathway signaling in cisplatin resistance [41, 42] and has particular relevance to HNSCC given recurrent pathway dysregulation observed in a sizeable subset of these tumors [51, 52]. Similarly, our data also opens critical questions as to the precise mechanistic role of recurrent *NOTCH* mutations in HNSCC, and what the functional impact of these alterations may be for tumors on response to chemotherapy. Indeed, we have optimized and validated a new genome scale functional genomics approach that may be one important tool for dissecting these mechanisms of cisplatin resistance in a larger set of models and adds to the growing data indicating that *NOTCH1* status may warrant formal evaluation as a potential predictive biomarker of cisplatin response. We posit that this approach will nominate additional targetable pathways that facilitate the clinical translation of strategies that target cisplatin resistance in HNSCC.

## Data Availability

The datasets used and/or analysed during the current study are available from the corresponding author on reasonable request.

## Abbreviations

CRISPR: clustered regularly interspaced short palindromic repeats
FDR: false discovery rate
FPKM: fragments per kilobase million
GeCKO: Genome-wide CRISPR knockout
GSEA: gene set enrichment analysis
GSI: gamma-secretase inhibitor
HNSCC: head and neck squamous cell carcinoma
HPV: human papillomavirus
NGS: next-generation sequencing
